# Heat Adaptation Benefits for Vulnerable groups In Africa (HABVIA): a study protocol for a controlled clinical heat adaptation trial

**DOI:** 10.1186/s12889-025-22757-6

**Published:** 2025-05-09

**Authors:** Michaela Deglon, Chad Africa, Larske Marit Soepnel, Thandi Kapwata, Ama de-Graft Aikins, Kweku Bedu-Addo, Guy Howard, Estelle Victoria Lambert, Dale Elizabeth Rae, Martha Sibanda, Christopher Gordon, Mark New, Lara Ruth Dugas

**Affiliations:** 1 African Climate and Development Initiative, University of Cape Town, Cape Town, South Africa; 2 Division of Epidemiology and Biostatistics, School of Public Health, University of Cape Town, Cape Town, South Africa; 3 Julius Global Health, Julius Center for Health Sciences and Primary Care, University Medical Center Utrecht, Utrecht University, Utrecht, The Netherlands; 4 Environment and Health Research Unit, South African Medical Research Council, Johannesburg, South Africa; 5 Regional Institute for Population Studies, University of Ghana, Accra, Ghana; 6 Department of Physiology, SMS, Kwame Nkrumah University of Science and Technology, Kumasi, Ghana; 7 University of Bristol, Cabot Institute for the Environment, Royal Fort House, Bristol, UK; 8 Research Centre for Health through Physical Activity, Lifestyle and Sport (HPALS), Department of Human Biology, Faculty of Health Sciences, University of Cape Town, Cape Town, South Africa; 9 School of Health and Medical Sciences, Faculty of Health, Engineering & Sciences, University of Southern Queensland, Toowoomba, Australia; 10 Slum Dwellers International, Cape Town, South Africa; 11 Institute for Environment and Sanitation Studies, University of Ghana, Accra, Ghana; 12 Public Health Sciences, Parkinson School of Health Sciences and Public Health, Loyola University Chicago, Maywood, IL USA

**Keywords:** Climate change, Adaptation, Heat exposure, Health impacts, Passive cooling, Controlled trial, Sub-Saharan Africa, South Africa, Ghana

## Abstract

**Background:**

Temperatures across Africa are expected to rise at up to twice the rate of mean global temperatures, posing significant health threats to vulnerable communities. Prolonged exposure to high day- and night-time temperatures has been implicated in a myriad of adverse health outcomes. The built environment and inadequate housing can exacerbate these consequences, prompting the need to evaluate heat adaptation interventions as a sustainable adaptation strategy for low-income and informal settlement dwellers. The Heat Adaptation Benefits for Vulnerable groups In Africa (HABVIA) study aims to assess the impact of passive cooling interventions in homes on several key physiologic and mental health outcomes, as well as building internal thermal conditions.

**Methods:**

HABVIA is a 3-year prospective controlled study to identify, implement and assess heat adaptation solutions in four low-income communities in one urban and one rural site in Ghana and South Africa, respectively. In each site, *N*=240 participants (*N*=60 per site) will be assigned to intervention or control groups. The intervention is focused on lowering the nighttime temperature of the home environment. Health and biometric data will be collected through a combination of physiological measurements, questionnaires, and biochemical measures taken at 3 time points during the hot season. Clinical outcomes include objective sleep behaviour, core body temperature, physical activity, blood pressure, blood glucose, anthropometrics, and body composition. Indoor and outdoor environmental data will be collected continuously using fixed indoor sensors and automatic weather stations. Housing and community characteristics, and socio-economic information will be collected. Quantitative comparisons will be made between intervention and control conditions using generalised linear mixed models. Qualitative data from consultive workshops will be used to assess the acceptability and feasibility of the adaptations.

**Discussion:**

Robust evaluation of the environmental and health outcomes of heat adaptations are limited for Africa, despite high climate vulnerability. HABVIA will address some of these gaps by assessing low-cost passive cooling interventions to promote heat resilience and improve health outcomes, providing real-world evidence for the feasibility of readily implementable and scalable adaptations in local contexts.

**Trial registration:**

Pan African Clinical Trials Registry (PACTR) PACTR202401521630856, version 1. Retrospectively registered on January 12, 2024.

**Supplementary Information:**

The online version contains supplementary material available at 10.1186/s12889-025-22757-6.

## Introduction

### Background and rationale

Temperatures across Africa are expected to rise at up to twice the rate of mean global temperatures, posing significant health threats to vulnerable communities [1]. Prolonged exposure to high day- and night-time temperatures has been implicated in a myriad of adverse health outcomes and has been associated with an increase in heat-related health risks across Sub-Saharan Africa (SSA) over recent decades [2]. Unfortunately, this trend is expected to do so even under the most ambitious emissions reduction scenarios [3, 4]. High ambient temperatures impact health both directly and indirectly [5–10]. Direct pathophysiological consequences include dehydration, increased burden on the cardiovascular system [11], and heat stroke, while warmer temperatures also indirectly impact health by increasing the risk of some infectious diseases and impacting food security [9]. Extreme heat has been associated with increased morbidity and mortality from conditions such as chronic kidney disease [8], respiratory disorders [6], adverse pregnancy outcomes [12], and compromised mental health conditions [13].

One proposed mechanism by which extreme temperatures may affect health outcomes is through disrupted sleep behaviour [14]. The role of sleep behaviour has recently gained traction as a key health indicator, underlying several biological functions implicated in the maintenance of cardiometabolic and mental health, immunity, and cognitive functioning [15]. Various dimensions of sleep behaviour, including sleep quality, sleep duration that is either too short or too long [16], mistimed sleep, and waking activity [17] have been associated with increased risk for the development of non-communicable diseases such as cardiovascular disease, obesity, diabetes, and depression [18–20].

The thermal environment also has important implications for sleep, as high temperatures can disrupt the thermoregulatory processes underpinning sleep onset and maintenance [21]. The effects of high radiant and air temperatures and humidity reduce the capacity for the body to cool itself via vasodilation and sweating [22], promoting wakefulness [23]. Additionally, residual effects of heat stress, cardiovascular strain and dehydration from exposure to high daytime temperatures can impact nocturnal decreases in core body temperature, disrupting sleep-wake cycles [9]. Notably, an emerging body of evidence suggests that higher outdoor or indoor temperatures are negatively associated with both sleep quality and quantity [24]. For example, a recent study pairing sleep observations from 68 countries and climate data found that increased night-time temperature delayed sleep onset and shortened sleep duration, thus increasing the probability of insufficient sleep [25]. These associations were stronger for residents of low-income countries and in hotter climates.

While humans are equipped with some capacity for physiological adaptation to heat exposure and changes in temperature through acclimatisation, this is limited by individual factors such as age, fitness-level, adiposity, and underlying chronic health conditions [26], as well as factors related to the external environment such as radiant temperature, humidity, and wind [22]. Achieving thermal comfort may therefore depend on heat adaptation interventions, such as passive cooling, that decrease exposure.

Passive cooling interventions that have successfully lowered indoor temperatures for occupants of urban informal settlements have been implemented in India, where various cool roof types have been shown to effectively reduce indoor ambient temperatures, compared to controls [27]. Regarding subsequent health outcomes, researchers in the United Kingdom have modelled the protective effect of external window shading to lower indoor temperature and decrease heat-related mortality risk [28]. In African settings, experimental testing of passive cooling interventions, using modelling methods and test cells, have included solar chimney height to maximise air velocity in Egypt [29], exterior coatings (texture and orientation) in Algeria [30], and solar shading, green roof, and reflective paints in Kenya [31].

While the results of these studies suggest a link between passive cooling interventions and reduced internal temperatures, the efficacy of these for improving human health outcomes has not been rigorously tested in non-laboratory conditions, in longitudinal cohorts, or using quantitative clinical measurements. Of the few reported heat adaptation interventions in SSA, many are modelling studies or are tested in artificial trial conditions and are not reflective of real-life circumstances [32, 33]. Therefore, robust evaluation of the environmental and health outcomes of heat adaptations in SSA Africa are critical, especially in real-world settings.

SSA, faces a widespread “adaptation deficit”, or lack of institutional, economic, and/or technological capacity to adapt to climate variability and weather extremes [34]. This adaptation deficit is juxtaposed against some of the hottest weather conditions across the globe, and a high extant burden of disease [35–37]. These factors disproportionally affect those living in informal and low-income settlements in SSA, where heat exposure is amplified due to urban heat island effects [38], inadequately cooled housing, and participation in livelihood activities and manual labour [39]. Climate services - easily accessible and disseminated climate information necessary for effective adaptation - are also sorely lacking in the region [40]. While there is growing evidence on the effectiveness of climate services in Africa [4, 41, 42], much of this is focused on drought, heavy rainfall, coastal and fluvial flood early warning. Heat early warning has received less attention. Although prototype heat early warning products are available [39] there has been little work on tailoring these products to the adaptation need and information access channels of specific vulnerable groups.

The lack of implemented heat adaptation in real-world settings in Africa poses a significant challenge to effective policy development and long-term climate adaptation planning for human health and well-being. This study primarily aims to explore the impact of passive cooling interventions in homes on key physiological and mental health outcomes, as well as on internal thermal conditions of buildings.

### Study aims and objectives

The HABVIA study aims to address some of the large evidence gaps in human health and wider social outcomes of heat adaptation in SSA, namely (i) the paucity of data from well-designed heat adaptation intervention trials in real-world high-vulnerability settings in the region and (ii) the absence of quantitative measurements of the health outcomes of these interventions. HABVIA aims to achieve this by identifying and implementing socially and culturally acceptable, affordable, and scalable heat adaptations for housing in low-income informal settlements in four communities in Ghana and South Africa.

Specifically, the following three objectives will be addressed:

- *Objective 1*. To explore the physiological response to extreme heat by quantifying the relationship between high seasonal ambient temperatures and humidity, and objectively measured health outcomes (primary: sleep; secondary: core body temperature, hydration) over three hot seasons in two rural and two urban African settings.
- *Objective 2*. To investigate any effects of passive-cooling interventions in lowering night-time indoor temperature and improving physical and mental health metrics, compared to controls.
- *Objective 3*. To determine the feasibility and social acceptability of such passive cooling adaptation interventions in climate vulnerable communities.

### Project team, partnerships and capacity building

HABVIA is part of the Wellcome Trust Heat Adaptation research programme aimed at testing and evaluating heat adaptation interventions to enhance public health in 11 low-and-middle income countries [43]. The collective efforts of the projects in this programme are designed to generate global evidence that will inform and support climate-health adaptation and policy development worldwide. Further HABVIA is collaborating with a diverse range of research partners including academic, governmental and policy makers as well as community outreach partners. The primary project team is a transdisciplinary team comprised of non-communicable disease and environmental health epidemiologists, human physiologists, climate scientists, heat-related policymakers, and climate service scientists ensuring a multidisciplinary approach to a complex problem. Underlying the success of HABVIA is the collaboration with often overlooked groups including a humanitarian/development non-governmental organisation, Slum Dwellers International. Capacity building of African health-climate researchers will be leveraged through the employment of post-graduate students, and the development of heat adaptation training seminars and field schools.

## Methods

The study methods will be reported in accordance with the Standard Protocol Items: Recommendation for Interventional Trials (SPIRIT) guidelines [44].

### Study design

HABVIA is a parallel-group controlled trial evaluating heat adaptation options in four sites, one urban and one rural low-income community each in Ghana and South Africa (SA), respectively. Participants will be assigned to intervention or control group using 1:1 block randomisation per site, with an equal distribution of male/females. An automated, computer-generated algorithm will be used for randomisation. Due to the logistical constraints of implementing adaptations in low-income settings, a building verification survey will be conducted to identify residences where the passive cooling interventions cannot be feasibly implemented. These participants will be automatically assigned to the control group and receive an alternative adaptation at the end of the study. Due to the nature of the interventions, participants and researchers will not be blinded to the intervention allocation. There are no pre-defined specific criteria for discontinuing the allocated interventions, but reasons for withdrawal will be tracked and reported in subsequent publications and taken into consideration for analysis.

### Setting

**Fig. 1:**
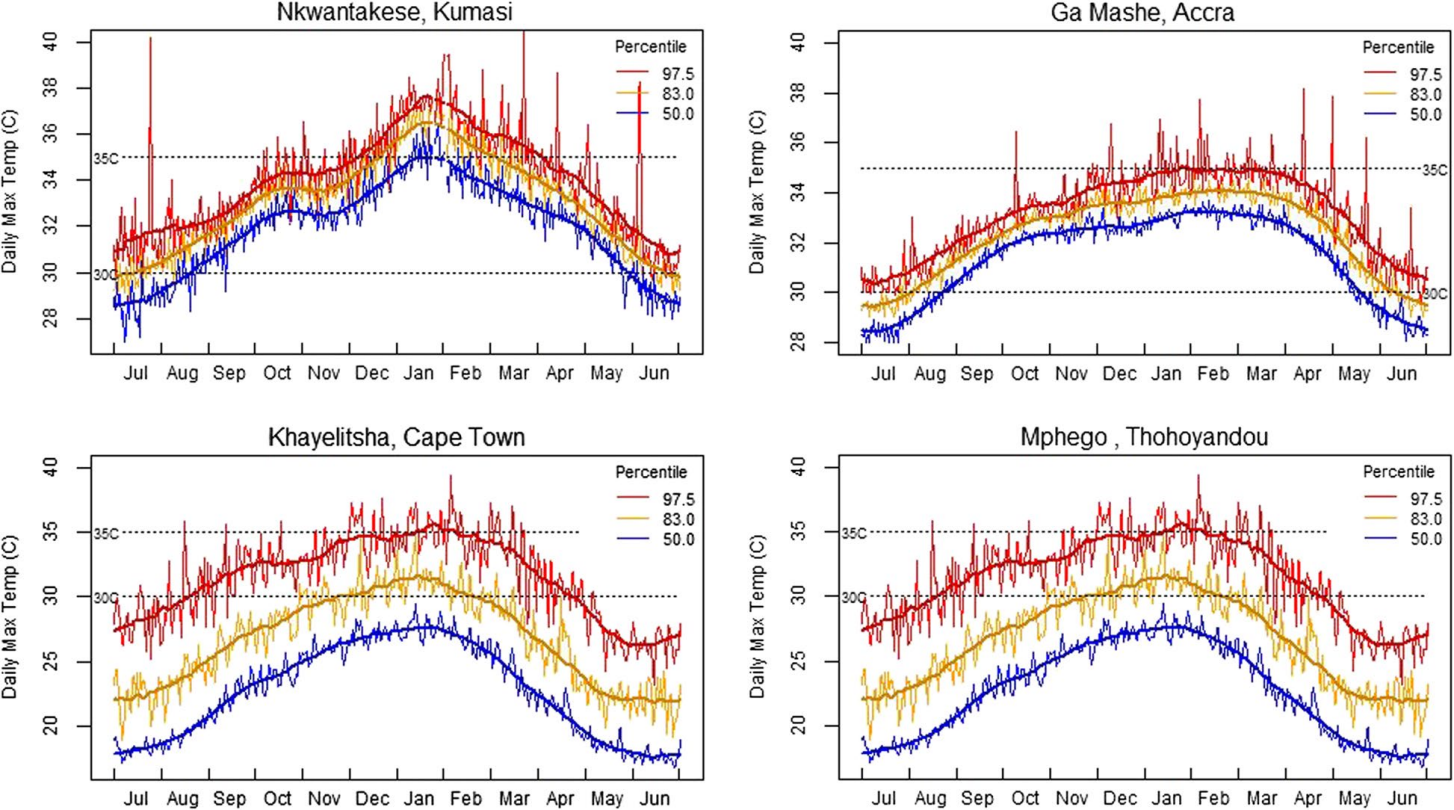
Extreme heat profiles for the four study sites, showing the daily maximum temperature exceeded approximately 2.5%, 17% and 50% of the time. Horizontal lines show two commonly used thresholds for moderate and extreme temperatures that have the potential to affect human health

The trial will take place in four locations across Ghana and SA: (i) Ga-Mashie (Ghana), a coastal fishing community in Accra; (ii) Nkwantakese (Ghana), a rural village about 25 km outside of Kumasi; (iii) Site B in Khayelitsha (SA), a mixed formal-informal township in the greater Cape Town metropolitan area; and (iv) Mphego Village (SA), a rural community near Thohoyandou, Limpopo Province (see Annex 1 for a more detailed description of the study sites). All these locations experience hot-season days with moderate (> 30 °C) or extreme (> 35 °C) temperatures, considered to be potentially harmful to human health (Fig. 1). In three of the four study communities there are existing long-term health monitoring programmes against which heat-health outcomes can be evaluated.

### Participants and enrolment

Participants were recruited from ongoing observational research activities from the following studies: 1) METS-Microbiome Study [45], being conducted in Nkwantakese, Kumasi, Ghana, and Khayelitsha, Cape Town, South Africa; 2) Heat, Health and Violence Study (Kapwata and Gibbs, unpublished) in Mphego Village, Thohoyondou, South Africa; 3) Urban Health and Poverty Study and the Tsui Anaa Project [46] in Ga-Mashie, Accra, Ghana. Participants must be over the age of 18 years and live in one of the designated study communities. The following exclusion criteria apply: individuals with current symptomatic infectious diseases (particularly those inducing fever); pregnant or lactating women; persons with conditions preventing normal physical activities, e.g., lower extremity disability, and persons unwilling to implement the adaptation interventions. During the regularly scheduled research visits for their respective ongoing studies, potential participants were given information about the HABVIA study. A short screening tool was used to assess eligibility and potential interest in participation. At each of the study sites, 60 participants (*N*= 30 intervention and *N*= 30 control) were enrolled prior to the baseline measurement period in year 1 with additional recruitment and enrolment occurring in year 2 to account for drop-out.

### Sample size calculation

Due to the limited available literature on heat adaptation interventions and objective measures of sleep, sample size was calculated based previous findings around (i) the impact of a passive cooling intervention (reflective paints) on ambient indoor temperature, and (ii) the expected effect size in terms of sleep associated temperature change. In a simulated test structure emulating a South African, urban informal settlement dwelling, Kimemia and colleagues [32] found that cooling paints lowered mean daily temperatures by 4.3 °C. At a significance of 0.05 and a power of 80%, this results in a sample size of *n*= 24 per group (intervention and control). Based on previous (observational) studies, an increase in temperature (4.3 °C) has been associated with a 11.6-minute decrease in sleep duration [47]. To account for the anticipated attrition rate over the three hot seasons we will aim to recruit 60 participants (*n*= 30 intervention and *n*= 30 control) at each of the site.

### Research procedures

**Fig. 2:**
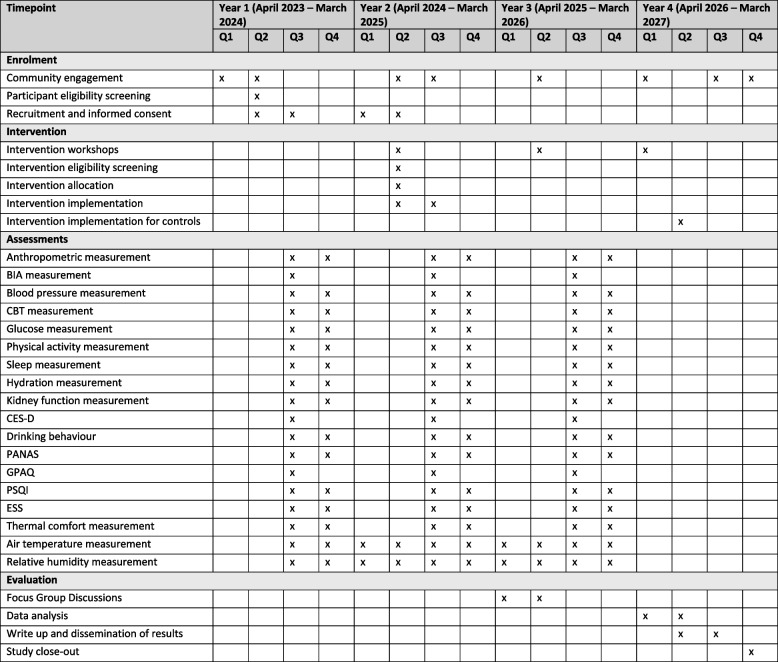
Schedule of enrolment, interventions, and assessments

An overview of the research activities is illustrated in Fig. 2.

**Fig. 3:**
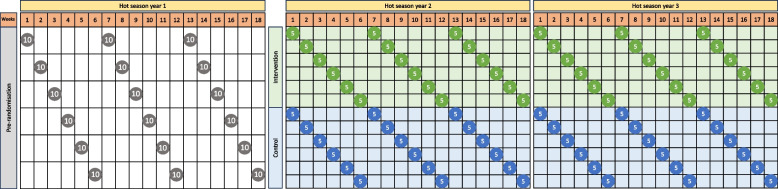
Measurement schema for physical health data collection over three hot seasons. There will be 60 subjects in each site, 30 intervention and 30 control. These will be split into 6 subgroups, each of which is monitored over three 7-day periods, evenly spaced over the hot season

The measurement of health outcomes will occur over three consecutive hot seasons, (November to March) with one baseline collection during the 2023 - 2024 hot season and two subsequent post-intervention data collection periods (2024 - 2025 and 2025 - 2026). The interventions will be identified by the study team and presented to the participants during a series of workshops following the baseline measurements in year 1. The adaptation will be implemented following the baseline data collection in year 1, and prior to the start of year 2 hot-season data collection period. For each hot season monitoring period, participants at each site will be grouped into six equal groups of *n*= 10 and monitored for three non-consecutive one-week periods, over 16-week hot season. We have accounted for approximately 4-weeks of suspended research activities during December and January months due to local holidays (Fig. 3). The 7-day health measurement period will include an initial clinic visit (lasting approximately 3 hours) followed by six consecutive 24-hour periods of off-site physiological data acquisition.

Throughout the 16-week hot season, indoor temperature and humidity data will be recorded continuously, while external climate data will be collected continuously throughout the year with the data being provided to the research team by various meteorological agencies, supplemented by project installed weather stations (also see Environmental data, below).

### Design and implementation of heat adaptation Interventions

At each study site, we will implement experimental heat-adaptation building modifications. The selection of these interventions will be informed by (i) findings of a scoping review of heat adaptations for homes which is currently being undertaken, (ii) local knowledge on heat stress conditions and potential adaptations; and (iii) assessment of affordability - critical to the scalability of interventions in low-income contexts, - technical feasibility, and social acceptability. By ensuring that the adaptations are appropriate and feasible in each context, we hope to establish early project buy-in from community and wider stakeholders such as local policy makers and governmental organisations, ultimately supporting climate resilience investments.

A series of workshops will be held prior to the intervention implementation and subsequently between each measurement period to obtain feedback and inform modifications. The workshops will include explanatory engagement around the passive cooling interventions. The purpose of which is to: 1) collate opinions, feedback, and gauge community buy-in, 2) build knowledge capacity of community residents around different adaptation options, 3) explore potential secondary and/or alternative adaptation options, and 4) to provide an equal and equitable platform for different stakeholders to share knowledge. Qualitative data from post-implementation workshops will inform assessments of the mechanisms of impact, based on United Kingdom Medical Research Council guidance for the evaluation of complex interventions [48]. This will include qualitative insights into intervention preferences and acceptability, as well as the wider socio-economic benefits and trade-offs that arise from the adaptations.

### Focus group discussions

Throughout the 3-year measurement period, a series of focus group discussions will be held to better understand the current and future challenges participants face during extreme heat. The discussions will be centred around two main topics 1) existing heat adaptation strategies participants may be employing in response to hot temperatures, and 2) climate literacy and knowledge of heat and other meteorological variables as they relate to health. The focus groups will consist of between 8–10 people and will be conducted throughout the study period to ensure equal representation of the cohort and allow for tracking of how the project changes climate-health literacy among its participants.

### Heat early warning

As an additional outcome the project team, in collaboration with the respective country national meteorological agencies, will co-design and trial heat early warning advisories and appropriate delivery routes for low-income and informal settlement dwellers. The advisories will be community orientated and targeted at informing both immediate and medium-term behavioural responses such as activity planning for hot weather days.

### Data collection

Data will be captured in the following areas: Health and physiological data; Environmental data; and Residential, community, and socio-demographic data. Table 1 details the study outcomes including the definition, functional form of the variable, measurement device, and collection timing. The data collection teams at each site will receive centralised training specific to the data collection procedures for this study. During the study period, field teams will regularly monitor the condition of the interventions and note any deterioration. Participant retention and adherence will be addressed with regular check-ins from the data collectors and reminders of the importance of wearing the devices.

**Table 1:** Health and environmental outcomes

**Outcome**	**Definition**	**Functional form**	**Device/tool**	**Timing of collection**
Clinical measures				
Anthropometrics	Weight (kg), height (m), waist circumference (cm)	Continuous	Seca 813 digital scale; stadiometer; measuring tape	Start of measurement week
Body composition	Body fat mass, body fat %, and fat free mass measured using BIA	Continuous	Quantum Legacy BIA Analyzer	Once per hot season
Blood pressure	Systolic and diastolic resting blood pressure (mmHg) measured in triplicate on two occasions	Continuous	Omron Automatic Digital Blood Pressure Monitor	Start of measurement week
Core body temperature	Internal body temperature (°C) estimated using thermal energy transfer	Continuous	CORE sensor	Continuous over 7-day period
Glucose	Fasting capillary blood glucose (mmol/L)	Continuous	AccuChek Instant Device	Start of measurement week
Physical activity	Acceleration counts per minute	Continuous	Actical accelerometer	Continuous over 7-day period
Sleep behaviour	Actigraphy-derived sleep behaviour (various parameters)	Continuous	Actiwatch Spectrum Plus	Continuous over 7-day period
Hydration status	Urine specific gravity, measure of number of solutes dissolved in urine as compared to water (1.000)	Continuous	Siemens Clinitek Status Analyzer	Start of measurement week
Kidney function	Albumin-to-creatinine ratios (mg/mmol categories)	Categorical	Siemens Clinitek Status Analyzer	Start of measurement week
Questionnaires				
Depression	Responses to 20-item questionnaire rating depression symptom (0 to 3 for each item); scores ranging 0–60, higher scores indicate greater depressive symptoms	Categorical	Center for Epidemiologic Studies Depression Scale (CES-D)	Once per hot season
Drinking behaviour	Self-reported average amount and frequency of liquid consumption	Count	Adapted food frequency questionnaire	Start of measurement week
Mood	Self-reported questionnaire containing 2 10-item scales (positive and negative affect scores between 10–50).	Categorical	Positive and Negative Affect Scale (PANAS)	End of measurement week
Physical activity	Self-reported physical activity participation in three settings and sedentary behaviour in the past week (MET-minutes)	Categorical	Global Physical Activity Questionnaire (GPAQ)	Once per hot season
Sleep quality	Self-reported sleep quality during the past month. Global PSQI score ranging from 0 to 21. Higher scores indicate poorer sleep quality	Categorical	Pittsburgh Sleep Quality Index (PSQI)	End of measurement week
Excess daytime sleepiness	Responses to 8 items rated on 4-point Likert scale, total score 0–24 with higher number indicating a higher daytime sleepiness	Categorical	Epworth Sleepiness Scale (ESS)	End of measurement week
Thermal comfort	Rating of nocturnal thermal comfort on a 7–point Likert scale	Categorical	ASHRAE 7-point thermal sensation scale	During each night of the measurement week
Indoor thermal conditions				
Air temperature	Ambient indoor air temperature (°C)	Continuous	DS1923 iButton Hygrochron heat and humidity measurement device	Continuous during the study period
Relative humidity	Water vapor present in the air compared with the total that can be held at a given temperature (%)	Continuous	DS1923 iButton Hygrochron heat and humidity measurement device	Continuous during the study period
External meteorological conditions				
Air temperature	Ambient outdoor air temperature (°C)	Continuous	Automatic Weather Station	Continuous during the study period
Relative humidity	Water vapor present in the air compared with the total that can be held at a given temperature (%)	Continuous	Automatic Weather Station	Continuous during the study period
Wind speed	Hourly average air speed (m/s)	Continuous	Automatic Weather Station	Continuous during the study period
Sunshine	Hourly and total sunshine hours per day	Continuous	Automatic Weather Station	Continuous during the study period
Rainfall	Hourly amount of rainfall (mm)	Continuous	Automatic Weather Station	Continuous during the study period
Cloud cover	Portion of the sky (octas) covered by all types of cloud at the time of observation	Count	Observation	Daily during the study period

#### Health data

The health data will be collected through a combination of physiological measurements, questionnaires, and biochemical measures over three measurement periods during the hot season each year. While the primary health outcome for HABVIA is sleep behaviour, other important health outcomes including core body temperature, hydration status, physical activity, blood pressure, fasting blood glucose, body composition, mental health and perception of thermal comfort will be measured.

##### Sleep behaviour

Participants will wear an Actiwatch Spectrum Plus (Philips Respironics, Bend, OR, USA) on their non-dominant wrist for 7 days which will include both work (typically weekdays) and at least one free (typically weekend day). Sleep data will be collected in 30-second epochs and analysed using Actiware software (v6.4, Philips Respironics, Bend, OR, USA). The following outcome variables will be derived: bedtime, sleep onset, sleep offset, sleep duration (total number of minutes scored as sleep), and mid-sleep time (mid-point between sleep onset time and wake time). Ambient 24-h light exposure throughout the 7 days will also be measured from the Actiwatch Spectrum on the wrist. Participants will be asked to keep the sensor uncovered at all times. Additionally, participants will be asked to keep a daily sleep log and press an event marker on the Actigraph at bedtime and wake-up time. Data will be downloaded and reviewed with each participant to clarify inconsistencies when the watch is returned. Rest intervals will be set using reported “try to fall asleep” times and wake up times on daily sleep logs or event markers if these times are missing. This sleep data collection methodology is based on a well-established protocol [49].

Subjective sleep assessments will be conducted at the end of each measurement week using the following questionnaires: 1) The Pittsburgh Sleep Quality Index (PSQI) [50], a validated and widely used tool to score sleep quality during the previous month. This tool comprises 19 questions related to sleep habits, the scores of which are summed for a global sleep quality score. Scored sleep components include subjective sleep quality, sleep latency, sleep duration, sleep efficiency, sleep disturbance, use of sleep medication, and daytime disfunction. A cut-point of 5 or less is used to denote good sleep quality. 2) Epworth Sleepiness Scale (ESS) [51], a screen for excessive sleepiness, namely average sleep propensity in “recent times”. Responses to eight questions are summed to obtain a total score (0–24), with higher scores indicating greater levels of daytime sleepiness. The questions refer to the likelihood of failing asleep during a range of activities with different somnificities. Both questionnaires have been used successfully in low-income communities in South Africa [49].

##### Core body temperature

Core body temperature will be measured continuously throughout the 7-day monitoring period using a small monitoring device (CORE sensor, Greenteg, Switzerland) equipped with a thermal energy transfer sensor that can measure and transmit internal body temperature data. According to the manufacturer [52], this device provides a valid measure of core body temperature, with an absolute mean deviation of 0.21 °C, a standard deviation of 0.28 °C, and a 95% confidence interval of ± 0.56 °C, when compared to an ingestible radio pill (E-Celsius Pill, Body Cap) in daily life and sports contexts. The monitor is placed directly on the skin and secured in place using a nylon chest strap. The preferred mounting position is on the left side of the torso (apical) about 20 cm below the armpit, directly on the ribcage between pectoral muscle and latissimus muscle. Participants will be instructed to always wear the monitor except when bathing or showering.

##### Hydration status

Hydration status will be measured at 3-time points during the hot season and estimated by means of a mid-stream urine sample using a Clinitek Status + Analyzer, a point-of-care urinalysis machine (Siemens, Munich, Germany). The analyzer provides automated reading of the Multistix® test strip, encompassing an array of assays (leukocyte, nitrite, protein, blood, glucose, ketone, bilirubin, urobilinogen, pH, specific gravity, creatinine, albumin). Albumin-to-creatinine ratios will additionally be calculated as an indicator for kidney function.

Behaviour around hydration will be assessed during each clinic visit using a questionnaire adapted from a culturally validated food frequency questionnaire [53]. Participants self-report average amount and frequency of liquid consumption.

##### Physical activity

Physical activity will be assessed objectively using the Actical accelerometer (Philips Respironics, Bend, OR, USA). The monitor will be worn at the waist, positioned just behind the left hip. Each participant will be asked to wear the activity monitor at all times during the 7-day monitoring period, including during sleep; the only time the monitor should be removed will be while bathing, showering, or swimming. The actical records accelerations (cpm) in 1-min epochs, which is used to estimate sedentary activity (< 100 cmp), moderate activity (1535 - 3959 cpm) and vigorous activity (≥ 3960 cpm) using established cut-points [54, 55].

Physical activity will additionally be assessed once per hot season by self-reported questionnaire, using the Global Physical Activity Questionnaire (GPAQ, version 2) [56]. The main outcome variables of the GPAQ include a categorical variable of total physical activity (high, moderate and low) and a continuous variable of physical activity within the domains of work, transport and leisure.

##### Blood pressure

Systolic and diastolic blood pressure and heart rate will be measured at the start of each measurement week using the Omron Automatic Digital Blood Pressure Monitor (model HEM- 907XL, Omron Healthcare, Bannockburn, IL, USA). Following a 5-minute resting period, and with the antecubital fossa at heart level, three measurements will be made on the right arm. This will be repeated approximately 60 minutes later, resulting in a total of 6 blood pressure readings. To calculate average systolic and diastolic blood pressure, the 1^st^ and 4^th^ measurements are discarded, with the remaining measurements being averaged. Blood pressure measurements are used to categorise hypertension, which includes either a systolic blood pressure ≥ 140 mmHg, diastolic blood pressure ≥ 90 mmHg, being told by a doctor that they have hypertension or using blood pressure medication [57].

##### Fasting blood glucose

Fasting blood glucose will be measured at the start of each measurement week using the AccuChek Instant point of care device. A drop of capillary blood will be collected from the participants’ non-dominant hand. Blood glucose measurements will be used to estimate type 2 diabetes risk using the International diabetes Federation cut-points; diabetes (> 7 mmol/l) and pre-diabetes (6.1–7.0 mmol/l) [58].

##### Anthropometrics and body composition

The participants will be weighed at the start of each measurement week, without shoes and dressed in light clothing, to the nearest 0.1 kg using a standard digital scale (model 813, Seca, SC, USA). Height will be measured to the nearest 0.1 cm using a stadiometer without shoes, with the participants’ head held in the Frankfort plane. The participants weight and height measurements will be used to calculate body mass index (BMI) as weight/(height)^2^, and assigned as normal weight (BMI < 25 kg/m^2^), overweight (25–29.9 kg/m^2^) and obese (≥ 30 kg/m^2^) [59]. Waist circumference will be measured to the nearest 0.1 cm at the umbilicus. Hip circumference will be measured to the nearest 0.1 cm at the point of maximum extension of the buttocks.

Body composition (% body fat) will be assessed once per hot season using bioelectrical impedance analysis (BIA, using BIA Quantum, RJL Systems, Clinton Township, MI), and study specific equations [60]. Laying in a supine position with limbs abducted, current-supplying electrodes will be placed on the dorsal surfaces of the right hand and foot at the metacarpals and metatarsals, respectively. Detection electrodes will be placed at the pisiform prominence of the right wrist and the anterior surface of the true ankle joint. The single-frequency instrument will be attached to electrodes and generate an excitation current of 800 μA at 50kHz. The body fat mass and fat-free mass will be calculated from an estimate of total body water which in turn is estimated from the measured impedance of body tissue.

##### Mental health

Dimensions of mental health will be captured using the following: 1) The Center for Epidemiologic Studies Depression Scale (CES-D) [61] once per hot season. This tool has been successfully validated in South African populations [62]. 2) The Positive and Negative Affect Scale (PANAS) [63] will be used to assess mood at the end of each monitoring week. These questionnaires will be translated into local languages and checked for understanding.

##### Thermal comfort

Subjective thermal comfort will be measured daily during the 7-day nocturnal rest period and captured using the ASHRAE [64] 7-point thermal sensation scale: 1(cold), 2 (cool), 3 (slightly cool), 4 (Neutral), 5 (slightly warm), 6 (warm), 7 (hot). A question is included in the sleep diary which participants will complete each night during the off-site monitoring period.

#### Environmental data

Indoor temperature and humidity will be recorded continuously at 30-minute intervals, using a fixed hygrochron sensor (model DS1923-F5# iButton, Maxium Integrated, CA, USA) installed in the main sleeping area. External meteorological data including temperature, humidity, wind speed, sunshine, cloud cover, and rainfall will be collected and analysed as a control for internal conditions as well as to establish long-term temperature (and other climate variables) statistics and trends.

For three of the study sites, this data will be provided by the South African Weather Services (SAWS) and Ghana Meteorological Agency who operate Automatic Weather Stations in the vicinity. For the remaining rural site in Kumasi, a cellular wireless weather station (model WS-WH- 6006, Ecowittt, HK) was installed at a height of 6.85 m above ground, in the village. The installation height was elevated above the standard 2 m [65] at the request of the chief, to mitigate the risk of potential damage. The wireless outdoor sensor includes a solar-powered multi-sensor array (rain gauge, thermo-hygrometer, wind direction/speed sensor, and UV light sensor), as well as an indoor temperature sensor which communicates using cellular network connection to a receiver unit. Data will be stored on an SD card.

#### Residential, community and sociodemographic data

Characteristics of each participant dwelling as well as the outdoor environment that might affect building temperatures (building density, landcover, nearby water bodies, trees, and other shading) will be surveyed and assessed using a combination of geospatial and ground survey data. Features of the residence that will be measured include dimensions, presence of windows and doors, materials of floors, walls, ceilings and roofs, and ventilation.

Key to the success of the heat adaptation interventions is an assessment of affordability and feasibility in local-contexts, therefore comprehensive socio-economic and demographic information will be collected and considered in the evaluation. A study-specific demographic and socio-economic survey which is currently being used in the ongoing studies, and has been shown to be culturally appropriate, will be employed.

### Data management

Quantitative data from multiple sources will be collated and stored in a secure relational database hosted at the University of Cape Town. Personal identification information will be removed, and data will be merged and stored using alphabetic-numerical identification codes to maintain participant anonymity. The data base will be password protected and available to the research team exclusively. Qualitative data will be stored as anonymised transcripts.

All environmental data will be archived on the University of Cape Town’s open data repository, ZivaHub [66], which is driven by Figshare. All data are easily locatable by searching within the platform, and through general internet searches. All datasets will have a persistent DOI which will be used to identify associated publications, raise awareness of the availability of the data, and facilitate searching. Project data will be available on request under a data sharing agreement that provides commitments to: 1) using the data only for research purposes and not to identify any individual participant; 2) securing the data using appropriate computer technology; and 3) destroying or returning the data after analyses are completed. After the project is complete, data will be freely available for use (CC-BY licence), except where there is ongoing analysis of the data for a specific output by the project team. All data will be fully accessible 12 months after the project completion, once all publications have been submitted. In the final year of the project, the HABVIA team will publish a short article, describing the dataset in Wellcome Open Research to help researchers discover, access, and reference the resource.

### Data analysis

For the statistical analysis, means and standard deviations will be presented for continuous variables; medians and interquartile ranges will be reported for continuous variables with asymmetrical distributions. Continuous variables with a small number of ordered categories, as well as all nominal variables, will be described with counts and percentages.

- Objective 1. The effect of ambient outdoor and indoor temperature on physiological health outcomes (primary: sleep; secondary: core body temperature, hydration) will be assessed both cross-sectionally and longitudinally using a univariable and multivariable generalized linear mixed model. For the multivariable model we will adjust for environmental conditions (humidity, cloud cover, wind speed, precipitation); health covariates (body composition, blood pressure, physical activity, glucose); participant characteristics (sex, age); household characteristics (roof and wall type; size) as well as differences between sites (country, rural vs. urban).
- Objective 2. The impact of the interventions will be tested in phases, first the association between intervention exposure and night-time indoor temperature, adjusting for household characteristics, site and intervention type. Secondly, the association between intervention exposure and sleep, our primary outcome as well as secondary physical and mental health outcomes. The mediating effect of night-time indoor temperature on this association will be investigated, while adjusting for the covariates described under Objective 1). In addition to this, structural equation modelling (SEM) will be used to produce a pictographic representation of a-priori determined relationships between the variables of interest. It does this by estimating path equations simultaneously allowing for the calculation of direct, indirect and total effects. For missing data, we will explore different imputation methods and run sensitivity analyses to explore whether participants lost to follow-up differ when compared to those who completed the study measurement periods.
- Objective 3. Qualitative data analysis will be done in parallel with the community engagement workshops and Focus Group Discussions, allowing emerging analysis to shape subsequent implementation and data collection procedures. The analysis will answer process evaluation questions and draw on various qualitative methods including but not limited to reflexive thematic analysis and codebook analysis. A qualitative analysis software (such as MAXQDA) will be used by the investigators to facilitate coding, data management, and data interpretation.

### Monitoring

An independent advisory committee has been appointed to provide project oversight. The committee is comprised of experts in human physiology, climate science, biostatistics, and investigators with expertise in clinical trials methodology. The role of the committee is to advise the research team with regards to the following criteria: (i) Study design to ensure participant safety, study conduct and progress, and (ii) recommendations regarding the continuation, modification and termination of the randomized controlled heat adaptation trial. The committee will meet annually throughout the study duration to review adherence to the protocol, performance of individual centres, and data quality and completeness.

### Ethics approval and dissemination

This protocol has been approved by the Ethics Committee and/or Institutional Review Board of each of the participating institutions (Human Research Ethics Committee, University of Cape Town (reference: 469/2023); Kwame Nkrumah University of Science and Technology Committee on Human Research Publication and Ethics (reference: CHRPE/AP/761/23); University of Ghana Noguchi Memorial Institute for Medical Research Institutional Review Board (reference: 024/23–24). The ethical review board will be notified of any protocol amendments that require their approval, and subsequently reported to all research staff and participants.

Written informed consent will be obtained for each participant enrolled in HABVIA. Informed consent documents as well as the clinical and non-clinical data collections will be conducted in the predominant local language of each area: Twi (Nkwantakese); Ga and Twi (Ga-Mashie); iXhosa (Khayelitsha), and Venda (Thoyondou). Project investigators and staff will be trained in research ethics, good clinical practice, and study procedures, and will thereby be equipped to explain the study in detail to all participants, review the informed consent documents, and answer any questions that may arise.

A series of cohort feedback meetings will communicate the study findings to participants and the community. Results of the project will be submitted to peer-reviewed journals and presented at both national and international conferences. Our data sharing policy will be included on our project website and highlighted at research talks. In the final year of the project, the HABVIA team will publish a short article describing the dataset in an open access publishing platform to maximise discoverability and promote reproducibility, transparency and impact.

## Discussion

Despite high vulnerability to heat-related health risk, few robust evaluations of the environmental, health and socio-economic outcomes of heat adaptations in real-world settings have been done in Africa. Indicating a dire need for reliable heat-health data and heat adaptations that are acceptable and feasible in local contexts. By addressing these gaps using extensive physiological measures, linked to environmental changes, the knowledge generated through HABVIA may help to inform policy to support the effective implementation of heat adaptations in sub-Saharan African settings, where they are sorely needed.

The controlled trial described in this protocol will explore whether passive cooling interventions impact on indoor ambient temperatures, particularly at night, and the extent to which they affect sleep quality and subsequent health outcomes. The limitations of this study include blinding issues, as it is not feasible to blind either participants or researchers to the intervention due to the highly consultive nature of the research as well as potential selection bias due to the leveraging of on-going cohorts. The study design is otherwise optimized for both internal and external validity by staggering monitoring periods, measuring potential confounders across health, climate and infrastructure, and by comparing group differences at both the cross-sectional and longitudinal endpoints.

As global warming progresses so will the need to build local capacity to respond and adapt to heat-related health issues in Africa. HABVIA aims to contribute to this by providing a multidisciplinary platform for climate-health research, education, and policy. In addition, the study’s commitment to community engagement for the design and implementation of the intervention, and the resulting mixed-methods approach, will allow for in-depth interpretation of the intervention outcomes and improve relevance and acceptability. Ultimately, the impact of these interventions will depend on uptake, by using locally sourced materials and generating site specific climate and social data that considers baseline health profiles, where pre-existing conditions may make people more vulnerable to heat stress, HABVIA is working towards tailored solutions for multi-vulnerable communities. This can also provide a framework for intervention development in future climate-related studies.

### Trial status

This clinical trial is registered with the Pan African Clinical Trials Registry (PACTR), trial ID PACTR202401521630856, version 1. Retrospectively registered on January 12, 2024. The research activities commenced in November 2023, with participant recruitment starting in December 2023 and concluding in September 2024. Research activities are planned to continue until March 2027. This version refers to version 1 (12^th^ January 2024) of the approved protocol.

## Supplementary Information

Supplementary Material 1.

## Data Availability

No datasets were generated or analysed during the current study.

## Abbreviations

AWS: Automatic Weather Station
BIA: Bioelectrical Impedance Analysis
CPM: Counts Per Minute
HABVIA: Heat Adaptation Benefits for Vulnerable groups In Africa
PACTR: Pan African Clinical Trials Registry
SSA: Sub-Saharan Africa
SA: South Africa
SAWS: South African Weather Services
UKRMRC: United Kingdom Medical Research Council
