# Supplementary material for: Heat Adaptation Benefits for Vulnerable groups In Africa (HABVIA): a study protocol for a controlled clinical heat adaptation trial
Source: BMC Public Health. 2025 May 9;25:1725. doi: 10.1186/s12889-025-22757-6 (PMC12063451; doi:10.1186/s12889-025-22757-6)
Supplement: Supplementary file 1 — Supplementary Material 1. [file 12889_2025_22757_MOESM1_ESM.pdf]

Annex 1: HABVIA Research Sites Description

HABVIA is capitalising on 4 ongoing longitudinal studies currently being conducted in two countries; Ghana and South Africa, with two research sites in each country, one of which is rural and the other is urban and high-density. The four research sites are described in detail below. Prof Lara Dugas is the principal investigator of “METS-Microbiome Study” being conducted in Nkwantakese, Ghana (rural) and Khayelitsha, South Africa (urban, high-density). Dr. Thandi Kapwata is the principal investigator of the “Heat, Health and Violence Study” in Mphego Village, Thohoyondou, South Africa (rural) and Prof Ama de-Graft Aikins is the principal investigator of the Regional Institute for Population Studies Urban Health and Poverty Study and the Tsui Anaa Project in Ga-Mashie, Ghana (urban, high-density).

Nkwantakese-Afigya Kwabre North District, Ashanti Region, Ghana.
The town is situated to the southwest of Agona Ashanti the District Capital and is about 20km from Kumasi with a population of approximately 5,000. The town is peri-urban, with approximately 600 households, built from a mixture of cement and mud brick and plastered with cement. The source of drinking water is generally boreholes. It is largely a rural economy that is extensively supported by a combination of small scale or subsistence farming and petty trading. The METS study operates from a brick building that was allocated by the village Chief. METS research participants are seen three days a week in the research clinic, which operates between the hours of 06h00 and 14h00. The research staff are employed by Kwame Nkrumah University of Science and Technology (KNUST) in Kumasi under the leadership of Professor Kweku Bedu-Addo.

Ga-Mashie, Accra, Ghana
Ga-Mashie is a coastal multi-ethnic community located in Accra, Ghana’s capital. The 2010 national census recorded a population size of 44,361, occupying an area of 242 acres. Population density is high, and the physical environment is heavily built up with poor housing structures and sanitary conditions. The main economic activities are fishing and petty trading. A large e-waste site, situated in the neighbouring Agbobloshie, provides a third source of livelihood. The community occupies the fourth and lowest income class within the Accra Metropolitan Area. Ga-Mashie faces a ‘double jeopardy’ of infectious and chronic conditions with serious health and development consequences. Climate-related events like heavy rainfall, seasonal flooding and high temperatures have been linked to a high incidence of malaria, cholera and non-cholera diarrhoeal disease, measles and cerebrospinal meningitis. A growing burden of non-communicable diseases, like diabetes and hypertension, is linked to an obesogenic and alcohol promoting environment. There is a high prevalence of psychosocial stress. Previous research identified heat as the third community stressor on a list of 16 stressors generated by community members. Other environmental stressors included heavy rains/floods and air/industrial pollution.

Site B, Khayelitsha, Cape Town, Western Cape, South Africa
The South African METS site is located at the David Flude Methodist Church, Site B, Khayelitsha, a high-density urban, informal township. It is the 3rd largest township in South Africa and is adjacent to the city of Cape Town. The exact size of the population is not known, the 2011 census counted 400,000 residents, but some estimates are closer to 2.4 million residents. Residents are overwhelmingly black and Xhosa speaking (>90%) and 70% of people live in shacks constructed from zinc metal sheets. The closest water access point is generally more than 200m away from the dwellings. 50% of residents are unemployed, and almost 90% of households report moderate or severe food insecurity. The METS research clinic operates three times a week between the hours of 08h00 and 14h00 and is currently following over 350 adults. The research staff are employed by the University of Cape Town under the joint leadership of Professor Estelle Lambert and Dr. Dale Rae.

Mphego Village, Thohoyandou, Limpopo Province, South Africa

Thohoyandou is a town in the Vhembe District which is in the Northern part of Limpopo Province. Limpopo is a predominantly rural province with over 80% of its population living in rural areas. Thohoyandou consists of small rural townships and villages. The town has a population of about 69 453 people and 17 345 households. Poverty is prevalent with more than half the population being dependent on government social grants for household income. Previous studies have shown that the Limpopo province is vulnerable to the environmental and health impacts of climate change due to its exposure to extreme weather events that include heatwaves and heavy rains. In addition, Thohoyandou was found to be one of the 10 hottest cities in the country based on simulations of the 99th percentile of maximum temperature from 1979 to 2009. Therefore, residents are at high risk of heat-related illnesses however they lack the resources to mitigate against heat impacts. The research site is run by Dr. Thandi Kapwata from the South African Medical Research Council. She is the lead investigator on the project exploring heat-related outcomes in over 400 households in Mphego Village, Thohoyandou.
